# Exploring Genetic Associations of Alzheimer’s Disease Loci With Mild Cognitive Impairment Neurocognitive Endophenotypes

**DOI:** 10.3389/fnagi.2018.00340

**Published:** 2018-10-30

**Authors:** Ana Espinosa, Begoña Hernández-Olasagarre, Sonia Moreno-Grau, Luca Kleineidam, Stefanie Heilmann-Heimbach, Isabel Hernández, Steffen Wolfsgruber, Holger Wagner, Maitée Rosende-Roca, Ana Mauleón, Liliana Vargas, Asunción Lafuente, Octavio Rodríguez-Gómez, Carla Abdelnour, Silvia Gil, Marta Marquié, Miguel A. Santos-Santos, Ángela Sanabria, Gemma Ortega, Gemma Monté-Rubio, Alba Pérez, Marta Ibarria, Susana Ruiz, Johannes Kornhuber, Oliver Peters, Lutz Frölich, Michael Hüll, Jens Wiltfang, Tobias Luck, Steffi Riedel-Heller, Laura Montrreal, Pilar Cañabate, Mariola Moreno, Silvia Preckler, Nuria Aguilera, Itziar de Rojas, Adelina Orellana, Montserrat Alegret, Sergi Valero, Markus M. Nöthen, Michael Wagner, Frank Jessen, Lluis Tárraga, Mercè Boada, Alfredo Ramírez, Agustín Ruiz

**Affiliations:** ^1^Research Center and Memory Clinic, Fundació ACE, Institut Català de Neurociències Aplicades, Universitat Internacional de Catalunya, Barcelona, Spain; ^2^Department for Neurodegenerative Diseases and Geriatric Psychiatry, University Hospital Bonn, Bonn, Germany; ^3^German Center for Neurodegenerative Diseases (DZNE), Bonn, Germany; ^4^Department of Psychiatry and Psychotherapy, University of Cologne, Cologne, Germany; ^5^Institute of Human Genetics, University of Bonn, School of Medicine and University Hospital Bonn, Bonn, Germany; ^6^Department of Genomics, Life & Brain Center, University of Bonn, Bonn, Germany; ^7^Department of Psychiatry and Psychotherapy, University of Bonn, Bonn, Germany; ^8^Department of Psychiatry and Psychotherapy, University Clinic Erlangen, Erlangen, Germany; ^9^Department of Psychiatry, Charité University Medicine, Berlin, Germany; ^10^Department of Geriatric Psychiatry, Central Institute of Mental Health, Medical Faculty Mannheim, University of Heidelberg, Mannheim, Germany; ^11^Center for Geriatric Medicine and Section of Gerontopsychiatry and Neuropsychology, Medical School, University of Freiburg, Freiburg, Germany; ^12^Department of Psychiatry and Psychotherapy, University of Göttingen, Göttingen, Germany; ^13^Department of Economic and Social Sciences & Institute of Social Medicine, Rehabilitation Sciences and Healthcare Research (ISRV), University of Applied Sciences Nordhausen, Nordhausen, Germany; ^14^Institute of Social Medicine, Occupational Health and Public Health, University of Leipzig, Leipzig, Germany

**Keywords:** Alzheimer’s disease, mild cognitive impairment, neurocognitive endophenotypes, genome-wide association studies, single-nucleotide polymorphism

## Abstract

The role of genetic risk markers for Alzheimer’s disease (AD) in mediating the neurocognitive endophenotypes (NEs) of subjects with mild cognitive impairment (MCI) has rarely been studied. The aim of the present study was to investigate the relationship between well-known AD-associated single-nucleotide polymorphisms (SNPs) and individual NEs routinely evaluated during diagnosis of MCI, AD, and other dementias. The Fundació ACE (ACE) dataset, comprising information from 1245 patients with MCI, was analyzed, including the total sample, amnestic MCI (aMCI) (*n* = 811), and non-amnestic MCI (naMCI) (*n* = 434). As probable-MCI (Pr-MCI) patients with memory impairment have a higher risk of AD, which could influence the statistical power to detect genetic associations, the MCI phenotype was also stratified into four related conditions: Pr-aMCI (*n* = 262), Pr-naMCI (*n* = 76), possible (Pss)-aMCI (*n* = 549), and Pss-naMCI (*n* = 358). Validation analyses were performed using data from the German study on Aging, Cognition, and Dementia in primary care patients (AgeCoDe), and the German Dementia Competence Network (DCN). SNP associations with NEs were calculated in PLINK using multivariate linear regression analysis adjusted for age, gender, and education. In the total MCI sample, *APOE*-ε4 was significantly associated with the memory function NEs “delayed recall (DR)” (β = -0.76, *p* = 4.1 × 10^-10^), “learning” (β = -1.35, *p* = 2.91 × 10^-6^), and “recognition memory” (β = -0.58, *p* = 9.67 × 10^-5^); and with “DR” in the aMCI group (β = -0.36, *p* = 2.96 × 10^-5^). These results were confirmed by validation in the AgeCoDe (*n* = 503) and DCN (*n* = 583) datasets. *APOE*-ε4 was also significantly associated with the NE “learning” in individuals classified as having Pss-aMCI (β = -1.37, *p* = 5.82 × 10^-5^). Moreover, there was a near study-wide significant association between the *HS3ST1* locus (rs6448799) and the “backward digits” working memory NE (β = 0.52, *p* = 7.57 × 10^-5^) among individuals with Pr-aMCI, while the *AP2A2* locus (rs10751667) was significantly associated with the language NE “repetition” (β = -0.19, *p* = 5.34 × 10^-6^). Overall, our findings support specific associations of established AD-associated SNPs with MCI NEs.

## Introduction

Alzheimer’s disease is the most common cause of dementia (Blennow et al., 2006), representing 50–60% of all cases. The risk of AD results from complex interactions of genetics, epigenetics, and environmental factors. In AD, the contribution of genetic factors to disease occurrence is estimated at up to 79% (Wingo et al., 2012). Since the advent of high-throughput genomics and GWASs, research into the genetics of AD has been highly successful in identifying risks factors for the disease. In 2013, the International Genomics of Alzheimer’s Project consortium analyzed >74,000 individuals, identifying 21 risk loci for AD besides *APOE*, 11 of which were novel susceptibility factors (Lambert et al., 2013); however, while such studies identified factors contributing to general susceptibility to AD, associations with specific endophenotypes, such as disease progression or cognitive functions, are less clear. For example, for disease progression to AD dementia, the only consistent association identified is with *APOE* (Elias-Sonnenschein et al., 2011; Lacour et al., 2016).

Endophenotypes are quantitative trait loci (QTLs) believed to be closely related to underlying disease pathophysiology. Hence, by using quantitative endophenotypes, rather than qualitative case/control status, as the phenotype for a genetic study, it is possible to reduce clinical diagnostic heterogeneity, thus increasing the power to detect genetic associations. In addition, this approach can provide more specific hypotheses for the biological pathways via which associated variants modulate disease progression. As a proof of principle of this strategy in AD, we and others have identified and replicated genetic factors by analyzing GWAS data together with biomarkers of AD, such as cerebrospinal fluid levels of amyloid-42, Tau, and phosphorylated Tau (Cruchaga et al., 2013; Louwersheimer et al., 2016). Crucially, the sample sizes required for these studies are several orders of magnitude smaller than those used in case/control designs.

Neuropsychological tests represent a good source of NEs for AD, particularly those related to episodic memory impairment (MacAulay et al., 2015; Ramanan et al., 2015; Ramanan and Saykin, 2015). Impairment of episodic memory is usually the earliest clinical symptom in AD dementia, and in the prodromal phase of AD dementia it is referred to as MCI (Petersen et al., 1999; Morris et al., 2001; Estévez-González et al., 2003; Grundman et al., 2004; Moulin et al., 2004; Dubois et al., 2010). Individuals classified as Pr-aMCI, that is, those with memory storage impairment (impaired recall and recognition) and an absence of comorbidities that could explain these cognitive deficits (e.g., cerebrovascular disease, anxiety, or depression), have an 8.5-fold increased risk of conversion to dementia (mainly AD), compared with those classified as having a Pss-naMCI condition (Delis et al., 1991; Petersen et al., 1999; Lopez et al., 2003; Petersen, 2004; Espinosa et al., 2013, 2017).

It is anticipated that the genetic factors associated with AD susceptibility will influence NEs during MCI. Hence, research into the NEs of individuals with MCI represents a potentially fruitful approach to identification of those with incipient AD, or at risk of developing AD. Although impaired NEs (e.g., episodic memory impairment) may be a reliable early manifestation of disease, the systematic investigation of the relationships between AD genetic risk factors and their endophenotypic expression remains in its infancy.

The aim of the present study was to investigate the association of AD-related genes with a comprehensive set of NEs routinely evaluated during the assessment of normal cognition and dementia. Using genetic association techniques and three independent MCI datasets (*n* = 2332 subjects), we explored associations between AD loci and neuropsychological measures obtained from subjects with MCI.

## Materials and Methods

### Participants

For the purpose of this study, MCI patients from the ACE cohort (*n* = 1245) (Espinosa et al., 2013; Lacour et al., 2016), consisting of 811 aMCI (64.5% female) and 434 naMCI (66.1% female) patients >48 years old, were recruited and assessed from 2006 to 2013 at the Memory Disorders Unit, Fundació ACE, Institut Català de Neurociènces Aplicades, Barcelona, Spain (Boada et al., 2014). All MCI diagnoses were assigned by consensus at a case conference attended by neurologists, neuropsychologists, and social workers. The inclusion and exclusion criteria for study participation have previously been described in detail (Espinosa et al., 2013). Briefly, at the time of enrollment, MCI subjects were classified into aMCI and naMCI, single or multiple domain, using Petersen’s diagnostic criteria (Petersen et al., 1999; Petersen, 2004), including subjective memory complaints, normal general cognition, preserved performance in activities of daily living, absence of dementia, and a measurable impairment in one or more cognitive functions. All MCI subjects had a clinical dementia rating (CDR) of 0.5 (Morris, 1993), were functionally literate, had no severe auditory or visual abnormalities (including glaucoma and cataracts), and had an available DNA sample.

In addition, all MCI patients from the ACE dataset were also stratified into four MCI phenotype-related conditions as follows: Pr-aMCI (*n* = 262) (64.5% female), Pr-naMCI (*n* = 76) (55.3% female), Pss-aMCI (*n* = 549) (64.5% female), and Pss-naMCI subtypes (*n* = 358) (68.4% female). This is similar to the classification used by Lopez et al. (2003), but extended to the naMCI groups (Espinosa et al., 2013). All MCI subjects were classified as Pss-MCI when there were comorbidities that could explain, or contribute to, cognitive deficits, and as Pr-MCI when there were none. Therefore, subjects were classified as having Pss-aMCI when there were psychiatric, neurological, or systemic illnesses (e.g., cerebrovascular disease, history of head trauma, encephalopathy, infectious diseases, or developmental disabilities) that could cause cognitive deficits. For the Pss-aMCI and Pss-naMCI subtypes, only those subjects with cerebrovascular disease and psychiatric disorders (anxiety or depression) were included. By contrast, patients were classified as having Pr-aMCI or Pr-naMCI if there were no neurological, psychiatric, or systemic illnesses that could explain their cognitive deficits (for more details, see Supplementary Figure S1).

### Neuropsychological Assessment

All MCI patients completed the NBACE (Alegret et al., 2012, 2013). This diagnostic procedure assesses eight cognitive functions, as follows:

(1) Orientation – temporal, spatial, and personal orientation.
(2) Attention and working memory – digit spans (forward and backward) subtests from the Wechsler adult intelligence scale – third edition (WAIS-III).
(3) Processing speed and executive function – the automatic inhibition subtest from the Syndrome Kurz Test (SKT); phonetic verbal fluency (words beginning with ‘P’ in 1 min); semantic verbal fluency (‘animals’ in 1 min); the similarities subtest from WAIS-III (abbreviated to the first 10 items).
(4) Language – repetition (two words and two sentences); verbal comprehension (to correctly execute two simple, two semi-complex, and two complex commands extracted from the Alzheimer’s disease assessment scale (ADAS) and the Barcelona test battery); an abbreviated 15-item Boston naming test.
(5) Verbal learning and memory – word list learning test from the Wechsler memory scale – third edition (WMS-III) (without using the interference list).
(6) Praxis – block design subtests from WAIS-III [abbreviated so that items 6–9 were scored only for accuracy (1 point) without a time bonus]; imitation praxis (four items); ideomotor praxis (four items).
(7) Visual gnosis – the Poppelreuter test, Luria’s clock test, and the 15-objects test.
(8) Global cognition – the Spanish version of the clock test.

Ideomotor praxis, repetition, and verbal comprehension variables were not included in the analysis because they were practically constant in amnestic and non-amnestic MCI groups.

### The German Study on Aging, Cognition, and Dementia in Primary Care Patients and the German Dementia Competence Network

Data from MCI patients of the German study on Aging, Cognition and Dementia in primary care patients (AgeCoDe) (*n* = 503 with 193 aMCI and 310 naMCI) and the German Dementia Competence Network (DCN) (*n* = 583 with 284 aMCI and 299 naMCI) were used for validation analyses. The AgeCoDe is a prospective cohort study that recruited 3267 non-demented individuals aged above 75 years, based on general practitioner registries in six German cities (Luck et al., 2007). Participants were followed for up to 11.5 years. The DCN cohort is a prospective multisite longitudinal observational study that recruited 812 MCI patients with a minimum age of 50 years, based on memory clinic patients with MCI or early dementia recruited from 14 German academic hospitals (Kornhuber et al., 2009). In both datasets, MCI was diagnosed according to Winblad criteria (Winblad et al., 2004) and had a CDR of 0.5 (Morris, 1993). In the DCN, MCI was operationally defined as one standard deviation (SD) deficit in any test from an extensive neuropsychological test battery, no major changes in activities of daily living (Bayer-ADL score < 4), and informant report of cognitive decline. In AgeCoDe, MCI was diagnosed based on a 1 SD deficit in cognitive performance, as measured by the SIDAM neuropsychological test battery (Zaudig and Hiller, 1995) and the absence of dementia. To further differentiate between aMCI and naMCI, a 1 SD deficit in the CERAD-DR test (Morris et al., 1989) and a 1 SD deficit in the SIDAM memory subscale (Zaudig and Hiller, 1995) were used to define amnestic deficit in AgeCoDe and DCN, respectively.

In DCN and AgeCoDe, the NEs used for validation of verbal learning and memory functions were the CERAD immediate and DR test as well as the CERAD word list recognition task. In addition the Wechsler memory scale logical memory test I and II was applied in the DCN cohort only. In AgeCoDe, the SIDAM memory subscale was only used for phenotype definition but not for the assessment of memory and learning ability.

### DNA Extraction, SNP Selection, and Genotyping

DNA was extracted from 2332 MCI samples from the ACE, AgeCoDe, and DCN studies using commercial methods. SNP selection was based on a review of the literature. Only those SNPs at loci identified as associated with AD by GWAS or meta-GWAS efforts were selected. Molecular genotyping methods and further information on SNP selection and quality controls are described elsewhere (Corder et al., 1993; Roses, 2010; Roses et al., 2010; Lacour et al., 2016).

### Statistical Analysis

A multivariate linear regression analysis adjusted by age, gender, and educational level was carried out among every SNP and the NEs using PLINK software (v. 1.07) (Purcell et al., 2007). This procedure was performed for both (i) the total MCI sample and the aMCI and naMCI groups, and (ii) the MCI stratified into four subtypes (Pr-aMCI, Pss-aMCI, Pss-naMCI, and Pr-naMCI). PLINK software (v. 1.07) (Purcell et al., 2007) was used to analyze genetic variation by calculating the allele frequencies in our populations, the polymorphism information content, the Hardy–Weinberg equilibrium, and deviations in allele frequencies between the study groups, using the Chi-square or Fisher’s exact test, and linkage disequilibrium (LD) with flanking markers (when needed). Data are graphically represented using heat maps, where individual *p*-values contained in a matrix are represented by color-coding indicating the statistical significance values calculated for SNPs and hierarchical NEs using R (The Project R for Statistical Informatics software) (v. 3.3.1)^1^. Further, Bonferroni’s correction was executed to adjust for multiple testing. This procedure was performed for both of the following:

(i) the total MCI sample and the aMCI and naMCI groups, using the following simplified formula:
  0.05/[3 MCI phenotype-related conditions (i.e., aMCI, naMCI, and MCI total sample) × 24 NEs × 41 SNPs (including *APOE*)] = 1.69 × 10-*E*^-5^,
(ii) the MCI stratified into four subtypes as follows:
  0.05/[5 MCI phenotype-related conditions (including MCI total sample) × 24 NEs × 41 SNPs (including *APOE*)] = 1.02 × 10-E^-5^.

Thus, only observed associations below *p* ≤ 10^-5^ were considered significantly associated. A trend toward association was assumed at *p* ≤ 10^-4^. Finally, significant associations in the MCI total sample and aMCI and naMCI groups from the ACE dataset (i) were validated using data from the AgeCoDe and DCN cohorts, using R software (v. 3.1.0)^2^. Here, no adjustment for multiple testing was performed. Significant associations in the MCI stratified into four subtypes (ii) were not validated in AgeCoDe and DCN datasets because Pr-/Pss-MCI stratifications were not available in these cohorts.

### Ethical Issues

The study protocol complied with national legislation and the Code of Ethical Principles for Medical Research Involving Human Subjects of the World Medical Association. Written informed consent was obtained from all participants prior to blood extraction. IRB approval was also obtained for this specific project (PI13/02434).

## Results

### Between Group Comparisons of Demographic, Clinical, and APOE-ε4 Data

A comparison of the demographic, clinical, and *APOE*-ε4 data between aMCI and naMCI groups from the ACE dataset is presented in Table 1. There were no statistically significant differences between aMCI and naMCI patients in education or gender; however, they did differ in age (*F* *=* 4.71; *p* = 0.030). The aMCI group obtained lower scores on the Hachinski Ischemia scale (HIS) than the naMCI group (*F* = 7.74; *p* = 0.006). The aMCI group also had a higher frequency of *APOE*-ε4 allele carriers (presence of at least one ε4 allele) than the naMCI group (χ^2^ = 16.25; *p* = 0.001).

**Table 1:** Comparison of demographic, clinical, and APOE-ε4 data between patients with aMCI and naMCI phenotypes from the ACE dataset.

	aMCI	naMCI	Statistic	*p*
*n* (%)	811 (65.1)	434 (34.9)		
Sex, female [*n* (%)]	523 (64.5)	287 (66.1)	0.33^∗^	0.563
Education in years [*n* (%)]			1.79^∗^	0.181
<8	638 (78.7)	327 (75.3)		
>8	173 (21.3)	107 (24.7)		
Age in years [mean (SD)]	76.3 (7.0)	75.4 (7.2)	4.71^#^	0.030
MMSE [mean (SD)]	24.9 (3.0)	27.0 (2.3)	158.77^#^	0.001
HIS [mean (SD)]	2.5/1.9	2.9/2.9	7.74^#^	0.006
*APOE*-ε4, presence of ε4 or ε4 [*n* (%)]	294 (36.3)	109 (25.1)	16.25^∗^	0.001


^a^MCI, amnestic mild cognitive impairment; naMCI, non-amnestic mild cognitive impairment; MMSE, mini-mental state examination; HIS, Hachinski Ischemia scale; APOE, apolipoprotein E; SD, standard deviation; ^*^X^2^; ^#^F.

Comparisons of demographic, clinical, and *APOE*-ε4 data among the four MCI subtypes (Pr-aMCI, Pss-aMCI, Pss-naMCI, and Pr-naMCI) from the ACE dataset are detailed in Supplementary Table S1. There were no statistically significant differences among the four MCI subtypes in age or gender; however, they did differ in education (χ^2^ *=* 17.87; *p* = 0.001). The Pr-aMCI and Pr-naMCI groups had lower HIS scores than the Pss-aMCI and Pss-naMCI subtypes (*F* = 11.12; *p* = 0.001). In addition, the *APOE*-ε4 allele showed a significant difference in distribution with the following order of subtypes: Pr-aMCI > Pss-aMCI > Pss-naMCI > Pr-naMCI (χ^2^ = 32.04; *p* = 0.001).

Comparison of demographic, clinical, and *APOE*-ε4 data between the aMCI and naMCI groups from the AgeCoDe and DCN datasets is detailed in Supplementary Tables S2A,B.

### Between Group Comparisons of Neuropsychological Data

Comparison of neuropsychological data adjusted by age between aMCI and naMCI groups from the ACE dataset is detailed in Table 2. Bonferroni *post hoc* multiple comparisons analyses revealed that there were statistically significant differences between aMCI and naMCI groups in all neuropsychological variables (Table 2).

**Table 2:** The ANCOVA comparing NE scores between aMCI and naMCI groups from the ACE dataset.

NE on NBACE	aMCI mean (SD)	naMCI mean (SD)	*F* (3,537)	Eta^2^	*p*-Values after Bonferroni’s correction for multiple testing aMCI vs. naMCI
**Global orientation**	13.78 (1.52)	14.55 (0.88)	93.68***	0.07	0.001
**Attention and working memory**					
Forward digits WAIS-III	6.58 (1.70)	6.58 (1.69)	7.26*	0.01	0.007
Backward digits WAIS-III	3.20 (1.51)	3.68 (1.57)	27.73***	0.02	0.001
**Executive function**					
SKT (time in seconds)	45.37 (20.73)	39.45 (14.72)	27.13***	0.02	0.001
SKT (errors)	4.62 (5.22)	3.05 (3.74)	30.42***	0.02	0.001
PVF	8.40 (4.19)	10.02 (4.38)	40.25***	0.03	0.001
SVF	11.11 (3.83)	13.29 (4.17)	90.53***	0.07	0.001
Similarities WAIS-III	7.84 (2.98)	9.03 (2.65)	48.50***	0.04	0.001
**Language**					
Visual naming (15-BNT)	12.57 (2.42)	13.51 (1.74)	51.42***	0.04	0.001
**Verbal learning and memory WMS-III**					
Learning	16.44 (4.88)	23.20 (5.06)	582.86***	0.32	0.001
Delayed recall	1.37 (1.43)	5.20 (1.77)	1870.57***	0.60	0.001
Recognition memory	18.65 (2.74)	21.96 (1.64)	283.37***	0.31	0.001
**Praxis**					
Block design	2.63 (1.38)	3.04 (1.20)	26.23***	0.02	0.001
Imitation	2.87 (1.22)	3.25 (1.04)	30.14***	0.02	0.001
**Visual gnosis**					
Poppelreuter test	8.82 (1.46)	9.30 (1.07)	35.68***	0.03	0.001
Luria’s clocks test	2.51 (1.23)	2.85 (1.04)	24.58***	0.02	0.001
15-Objects test	10.75 (2.65)	11.48 (2.06)	5.22*	0.02	0.023
**Global cognition**	4.80 (2.17)	5.66 (1.75)	50.02***	0.04	0.001


*NE, neurocognitive endophenotype; NBACE, neuropsychological battery of Fundació ACE; aMCI, amnestic mild cognitive impairment; naMCI, non-amnestic mild cognitive impairment; global orientation, sum of temporal + spatial + personal orientations; WAIS-III, Wechsler adult intelligence scale, third edition; SKT, automatic inhibition Syndrome Kurz test; PVF, phonemic verbal fluency; SVF, semantic verbal fluency; 15-BNT, Boston naming test with 15 visual items; WMS-III, Wechsler memory scale, third edition; Learning (Trials 1 + 2 + 3 + 4); Recognition memory, correct answers; Block design, WAIS-III; Visual gnosis (responses); global cognition, clock test; SD, standard deviation; NS, *p* ≥ 0.05; ^∗^*p* < 0.05; ^∗∗^*p* ≤ 0.005; ^∗∗∗^*p* ≤ 0.001.*

In addition, comparisons of neuropsychological data among the four MCI subtypes from the ACE dataset adjusted by education are detailed in Supplementary Table S3. Only global orientation, learning, DR, and recognition memory (WMS-III) survived Bonferroni’s correction for the total number of tests performed (see Supplementary Table S3).

Comparison of neuropsychological data adjusted by education and age between the aMCI and naMCI groups from the AgeCoDe and DCN datasets is detailed in Supplementary Tables S2C,D.

### Genetic Associations With NEs in the Total Sample, aMCI, and naMCI Groups From the ACE Dataset

**FIGURE 1:**
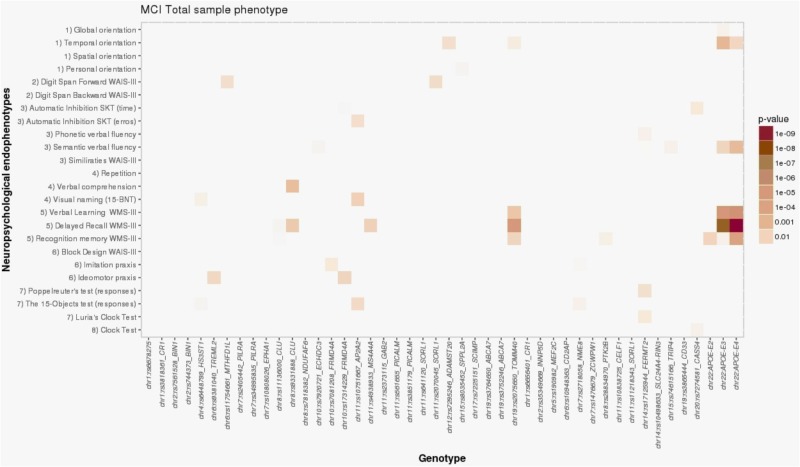
Heat map visualization of associations between AD-associated SNPs and NEs of individuals with MCI, including (1) orientation, (2) attention and working memory, (3) processing speed and executive functions, (4) language, (5) verbal learning and memory, (6) praxis, (7) visual gnosis, and (8) global cognition. Global orientation, summary of temporal + spatial + personal orientations; WAIS-III, Wechsler adult intelligence scale, third edition; SKT, Syndrome Kurz test; 15-BNT, the abbreviated Boston naming test with 15 items; verbal learning WMS-III, 1st + 2nd + 3rd + 4th trial scores; WMS-III, Wechsler memory scale, third edition.

In the total MCI sample, the *APOE*-ε4 allele was significantly associated with memory components of the WMS-III among NBACE NEs, including learning (L) (β = -1.35; *p* = 2.91 × 10^-6^), DR (β = -0.76; *p* = 4.10 × 10^-10^), and recognition (RE) (β = -0.58; *p* = 9.67 × 10^-5^) (Table 3 and Figure 1).

**Table 3:** Genetic associations with NEs in the total sample, aMCI, and naMCI groups from the ACE dataset.

MCI group	3A MCI total sample			3B aMCI			3C naMCI		
NE	NBACE-L	NBACE-DR	NBACE-RE	NBACE-L	NBACE-DR	NBACE-RE	NBACE-L	NBACE-DR	NBACE-RE
*N*	1243	1243	1243	809	809	809	434	434	434
β	–1.35	–0.76	–0.58	–0.80	–0.36	–0.36	–0.12	–0.23	0.17
L95/U95	–1.95/-0.76	–1.00/-0.51	–0.88/-0.27	–1.37/-0.23	–0.53/-0.18	–0.71/-0.02	–1.07/0.82	–0.56/0.09	–0.16/0.49
*p*-Value	2.91 × 10^-6∗^	4.10 × 10^-10∗^	9.67 × 10^-5^*^∗^*	0.004	2.96 × 10^-5∗^	0.029	0.792	0.138	0.300


*aMCI, amnestic mild cognitive impairment; naMCI, non-amnestic mild cognitive impairment; NE, neurocognitive endophenotype; NBACE, neuropsychological battery of Fundació ACE; NBACE-L, learning on memory assessed according to WMS-III within NBACE; NBACE-DR, delayed recall on memory assessed according to WMS-III within NBACE; NBACE-RE, recognition on memory assessed according to WMS-III within NBACE; β, unstandardized regression coefficient; L95/U95, lower/upper 95% confidence intervals. ^∗^Statistically significant after Bonferroni’s correction (*p* ≤ 1.69 10-*E*^-5^).*

The association of the NBACE-DR NE with *APOE*-ε4 in the total MCI sample was additionally supported by association with a SNP in strong LD with *APOE*, rs2075650 (*TOMM40* locus, β = -0.64; *p* = 9.16 × 10^-6^) (Figure 1; for details, see Supplementary Data available at: http://detritus.fundacioace.com/pub/supp_data/).

Additional nominal associations with NEs were detected for other AD susceptibility-associated SNPs in the total MCI sample (Figure 1). For example, the *APOE*-ε4 allele showed nominal association with the NBACE-temporal orientation NE (β = -0.16; *p* = 0.001). Additionally, significant associations were detected between a SNP at the *CLU* locus (rs9331888) and the NBACE-DR (β = -0.31; *p* = 0.006) and NBACE-verbal comprehension NEs (β = -0.07; *p* = 0.003).

**FIGURE 2:**
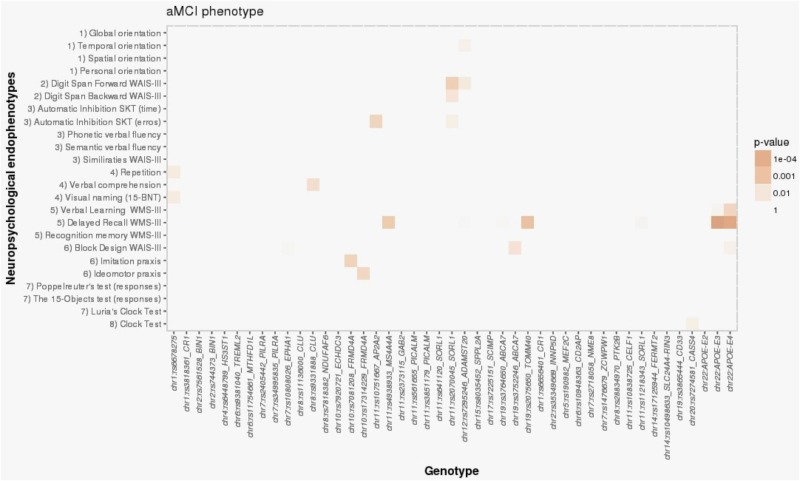
Heat map visualization of associations between AD-associated SNPs and NEs of individuals with aMCI, including (1) orientation, (2) attention and working memory, (3) processing speed and executive functions, (4) language, (5) verbal learning and memory, (6) praxis, (7) visual gnosis, and (8) global cognition. Global orientation, summary of temporal + spatial + personal orientations; WAIS-III, Wechsler adult intelligence scale, third edition; SKT, Syndrome Kurz test; 15-BNT, the abbreviated Boston naming test with 15 items; Verbal learning WMS-III, 1st + 2nd + 3rd + 4th trial scores; WMS-III, Wechsler memory scale, third edition.

For the aMCI group, the only significant association was between *APOE*-ε4 and the NBACE-DR NE (β = -0.36; *p* = 2.96 × 10^-5^) (Table 3 and Figure 2). Also, a nominal association was detected between the NBACE-DR NE and rs20175650 (*TOMM40* locus, β = -0.34; *p* = 0.001) (Figure 2).

For the naMCI group, there were no statistically significant associations between *APOE*-ε4 and any of the NBACE NEs (Table 3 for more details see Supplementary Data available at: http://detritus.fundacioace.com/pub/supp_data/).

### Genetic Associations With NEs in the Four Stratified MCI Subtypes From the ACE Dataset

In the Pss-aMCI group, *APOE*-ε4 was only significantly associated with the NBACE-L NE (β = -1.37; *p* = 5.86 × 10^-5^) (Supplementary Table S4B).

In the Pr-aMCI group, other AD loci, not linked to *APOE*-ε4, were detected (see Supplementary Table S5 and Supplementary Figure S2). The *HS3ST1* locus (rs6448799) exhibited a near study-wide significant association with the NBACE-backward digits NE (β = 0.52; *p* = 7.57 × 10^-5^). Also, the *AP2A2* locus (rs10751667) showed a significant association with the NBACE-repetition NE (β = -0.19, *p* = 5.34 × 10^-6^).

All of these associations survived Bonferroni’s correction (for more details, see Supplementary Data available at: http:// detritus.fundacioace.com/pub/supp_data/). The remaining AD-associated SNP markers were not significantly associated with any NE investigated in MCI subjects from the ACE dataset.

### Validation of Genetic Associations With NEs in the Total Sample, and aMCI and naMCI Groups From the AgeCoDe and DCN Datasets

In the total MCI sample from the AgeCoDe dataset, the *APOE*-ε4 allele exhibited significant associations with the NEs CERAD-IR (β = -1.14; *p* = 0.002), CERAD-DR (β = -0.41; *p* = 0.052), and CERAD-RE (β = -1.77; *p* = 0.051) (Table 4). In the DCN dataset, the *APOE*-ε4 allele was significantly associated with the WMS-III NEs LM-IR (β = -2.32; *p* = 1.85 × 10^-6^) and LM-DR (β = -2.70; *p* = 3.00 × 10^-7^), and with CERAD-RE (β = -1.69; *p* = 0.017) (Table 4).

**Table 4:** Genetic associations with NE in the total sample, aMCI, and naMCI groups from AgeCoDe and DCN datasets.

AgeCoDe	4A MCI Total sample			4B aMCI			4C naMCI		
NE	CERAD-IR	CERAD-DR	CERAD-RE	CERAD-IR	CERAD-DR	CERAD-RE	CERAD-IR	CERAD-DR	CERAD-RE
*N*	499	495	499	191	187	192	308	308	307
β	–1.14	–0.41	–1.77	–1.10	–0.32	–2.32	–0.52	–0.14	–0.08
L95/U95	–1.88/-0.41	–0.83/0.004	3.55/0.003	–2.08/-0.11	–0.90/0.25	–4.96/0.31	–1.55/0.50	–0.68/0.41	–2.47/2.31
*p*-Value	0.002	0.052	0.051	0.030	0.027	0.085	0.317	0.623	0.945

**DCN**	**4D MCI total sample**			**4E aMCI**			**4F naMCI**		
**NE**	**LM-IR**	**LM-DR**	**CERAD-RE**	**LM-IR**	**LM-DR**	**CERAD-RE**	**LM-IR**	**LM-DR**	**CERAD-RE**

*N*	556	556	550	274	274	272	282	282	278
β	–2.32	–2.70	–1.69	–2.29	–2.78	–1.73	–1.51	–1.41	0.26
L95/U95	–3.27/-1.38	–3.73/0.52	–3.08/-0.30	–3.42/-1.15	–4.04/-1.51	–3.84/-0.11	–2.99/-0.03	–2.92/0.09	–0.86/1.39
*p*-Value	1.85 × 10^-6∗^	3.00 × 10^-7^*^∗^*	0.017	9.9 × 10^-5^	2.18 × 10^-5^	0.110	0.046	0.077	0.643


*NE, neurocognitive endophenotype; aMCI, amnestic mild cognitive impairment; naMCI, non-amnestic mild cognitive impairment; CERAD, the consortium to establish a registry for Alzheimer’s disease; CERAD-IR, CERAD-immediate recall; CERAD-DR, CERAD-delayed recall; CERAD-RE, CERAD-recognition; LM-IR, logical memory immediate recall of the story from the Wechsler memory scale; LM-DR, logical memory delayed recall of the story assessed according to the Wechsler memory scale; β, unstandardized regression coefficient; L95/U95, lower/upper 95% confidence intervals. ^∗^Statistically significant after Bonferroni’s correction (*p* ≤ 0.05).*

In the aMCI group from the AgeCoDe dataset, *APOE*-ε4 was only significantly associated with CERAD-IR (β = -1.10; *p* = 0.030), CERAD-DR (β = -0.32; *p* = 0.027), and CERAD-RE (β = -2.32; *p* = 0.085) (Table 4). In the DCN dataset, the *APOE*-ε4 allele was significantly associated with the WMS-III NEs LM-IR (β = -2.29; *p* = 9.9 × 10^-5^) and LM-DR (β = -2.78; *p* = 2.18 × 10^-5^) (Table 4).

In the naMCI group from the AgeCoDe and the DCN datasets, *APOE*-ε4 was not statistically significantly associated with any of the NEs (Table 4).

Of note, *APOE*-ε4 was also associated with orientation in the ADAS in the total MCI DCN dataset (β = 0.31; *p* = 2.03 × 10^-7^). This association was additionally supported by a SNP in strong LD with *APOE*, rs2075650 (*TOMM40* locus; β = 0.26; *p* = 4.53 × 10^-6^) (for more details, see Supplementary Data available at: Supplementay Data Sheet S1 and Supplementary Tables S7–S9).

## Discussion

This study explored the relationship between well-known AD-associated SNPs and individual NEs obtained from subjects with MCI. We used a two-stage analysis, with a discovery and validation stage for the analysis. Our central goal was to determine if the variants associated with AD risk were also associated with NEs in individuals with MCI. However, our main finding is that except *APOE*, most of the other locus are not associated with NEs. Specifically, *APOE*-ε4 showed significant associations with performances in DR in the aMCI group, the Pss-aMCI group showed association between *APOE*-ε4 and performances in verbal learning and the Pr-aMCI group showed significant association between *AP2A2* and repetition and *HS3ST1* and backward digits. While several identified genes showed significant association with different phenotypes of MCI, including *APOE* gene, in contrast some genes such as *CLU*, failed which previous studies showed significance (Lacour et al., 2016).

In AD, neurocognitive functions, especially memory-related functions, are progressively affected, inexorably leading AD patients to become completely dependent on other people. For this investigation, we hypothesized that known genetic risk factors for AD may modulate specific neurocognitive functions in MCI. Consistent with our hypothesis, we identified a significant association between memory function and the *APOE*-ε4 allele, which is the strongest risk factor for AD. Specifically, the presence of the *APOE*-ε4 allele was significantly associated with learning, DR, and recognition memory function NEs in the total MCI sample, and with the DR NE in the aMCI group. These findings were confirmed by validation in the AgeCoDe and DCN datasets, and support previous observations that the *APOE*-ε4 allele is associated with increased memory dysfunction in patients with MCI close to conversion to AD dementia (Petersen et al., 1995; Smith et al., 1998; Bartres-Faz et al., 2001; Wilson et al., 2002; Farlow et al., 2004; Albert et al., 2007; Ramakers et al., 2008; Boyle et al., 2010). These NEs can be considered to represent the expression of complex neuronal networks, and their disappearance can already be detected in pre-dementia stages of AD (i.e., MCI). No other statistically significant associations were observed in either the complete MCI dataset or the aMCI or naMCI subgroups. This dearth of findings could potentially be interpreted as genuine lack of power of the present dataset for detecting very small effects. It is also possible that the AD risk loci identified to date have only subtle effects on MCI NEs in isolation. If this was the case, the actual effects of selected loci could be detected only by studying larger series, or via large meta-analyses, similar to case–control studies searching for AD risk genes (Lambert et al., 2013). This interpretation should be tested using larger series and subsequent meta-analyses in the future.

The *TOMM40* locus was only associated with the DR memory function NE in the total MCI sample. A previous study (Cervantes et al., 2011) showed that, among genetic variants in the *APOE* cluster region, *TOMM40* SNPs (Roses, 2010; Roses et al., 2010) were associated with progression from the MCI stage to AD (rs59007384 and rs11556510), as well as with shorter time to progression from MCI status to AD (rs10119); however, these results could not be replicated in independent cohorts. Interestingly, the *TOMM40* and *APOE* variants have been previously reported as significantly influencing age-related memory performance independently of one another (Caselli et al., 2009). When *APOE*-ε4 is excluded from the analysis, *TOMM40* appears to be a factor associated with risk of poor performance on the DR memory function NE in the MCI total sample. Accordingly, we detected some associations with rs2075650 (*TOMM40*) that closely resembled the findings for *APOE*-*ε4*; however, conditional analysis using epsilon 4 carrier status as a covariate for rs2075650-*TOMM40* resulted in no significant associations (*p* > 0.05) with any NE under study. This *post hoc* analysis suggests that the detected associations with *TOMM40* in this dataset were strictly related to its LD with the *APOE* locus. Similar results were obtained by conditional analysis using epsilon 4 carrier status as a covariate for rs2075650-*TOMM40*, with no significant associations (*p* > 0.05) with the orientation NE in patients with MCI from the DCN dataset.

Regarding the four stratified MCI phenotype-related conditions, we confirmed our previous observation (Espinosa et al., 2013) that the *APOE*-ε4 allele showed a significant difference in distribution with the following order among subtypes: Pr-aMCI > Pss-aMCI > Pss-naMCI > Pr-naMCI. According to a previous study (Quintino-Santos et al., 2015), the aMCI groups with the lower scores on memory dimension of the MMSE had the major presence of the *APOE-ε4*. Additionally, we previously reported an association between the presence of at least one *APOE*-ε4 allele at baseline and DR neuropsychological performance among Pr-aMCI, Pss-aMCI, Pr-naMCI, and Pss-naMCI groups (Espinosa et al., 2013); however, the presence of the ε4 allele was only associated with the learning memory function NE for the Pss-aMCI phenotype, that is, for those MCI subjects with memory impairment and comorbidities, such as anxiety, depression, or cerebrovascular disease, that could explain their cognitive deficits. Our results are not consistent with those of a previous study (Mackin et al., 2013), which found a higher frequency of ε4 allele carriers among MCI patients with subsyndromal symptoms of depression, but no association with poorer cognitive function based on evaluation according to the ADAS (Rosen et al., 1984).

Of note, we identified a near study-wide significant association between *HS3ST1* and working memory for the Pr-aMCI phenotype, a MCI subtype with enrichment for AD in pre-dementia stages (Espinosa et al., 2013, 2017). The *HS3ST1* locus exhibited a negative correlation on working memory function measured via the backward digits NE in Pr-aMCI patients (i.e., those MCI patients with memory impairment and without comorbidities, such as anxiety, depression, or cerebrovascular disease, that could explain their cognitive deficits) (Petersen et al., 1999; Lopez et al., 2003; Petersen, 2004; Espinosa et al., 2013, 2017). Interestingly, *HS3ST1* is a newly discovered AD locus (Desikan et al., 2015), and its gene expression is altered in AD compared with control brains. The previous study also demonstrated a genetic overlap between AD, C-reactive protein, and plasma lipids (i.e., triglycerides, and high- and low-density lipoprotein levels). The SNPs selected in our study were actually based on the original paper by Lambert et al. (2013). In that case, risk allele (OR: 1.08) was the allele T. In the present study the risk allele has also a positive association between *HS3ST1* locus with better scores in the “backward digits” working memory NE among individuals with Pr-aMCI. As we selected the genetic markers from Lambert et al. (2013) unfortunately we do not have the genetic markers proposed by Desikan et al. (2015) to make a direct comparison. Importantly, working memory impairment becomes more pronounced during MCI, and the very early stages of AD are also marked by working memory impairment, as well as by executive dysfunction and episodic memory deficits. These cognitive deficits begin during the MCI stage and appear to be a sign of progression to AD (Kirova et al., 2015). A negative correlation of HS3ST1 associated to working memory in individuals with the Pr-aMCI phenotype was observed in this study, and independently supports the role of this locus in AD pathogenesis; however, this finding is absolutely novel and requires independent validation in other series for confirmation. Hence, our results regarding *HS3ST1* must be interpreted cautiously at present.

Finally, *AP2A2* was associated with inferior language function performance, as assessed by the repetition NE, in patients with Pr-aMCI. Conversely, *AP2A2* locus showed a protective effect for the minor allele (A-allele) in the original study (Lambert et al., 2013). In contrast, we have detected a poorer performance associated with the language NE “repetition” among individuals with Pr-aMCI A-allele carriers. In addition, to determine the relationship between the genetic markers and memory impairment of hippocampal type, which is the pattern of memory impairment described in the literature as a highly suggestive phenotype of AD (Sarazin et al., 2007; Teichmann et al., 2017; Dubois, 2018), those individuals with Pr-aMCI and memory storage impairment (storage subtype) (Delis et al., 1991; Petersen et al., 1999; Lopez et al., 2003; Petersen, 2004; Espinosa et al., 2013, 2017) were included in an additional analysis. We found that *AP2A2* locus (rs10751667) was significantly associated with the language NE “repetition” for both, the Pr-aMCI (β = -0.19, *p* = 5.34 × 10^-6^) and for the Pr-aMCI-storage subtype (β = -0.24, *p* = 1.16 × 10^-6^) (Supplementary Figure S3 and Supplementary Tables S6, S10; for details see Supplementary Data available at: http://detritus.fundacioace.com/pub/supp_data/). Remarkably, a recent proteomic analysis identified dysfunction in cellular transport, energy, and protein metabolism in different brain regions of individuals with atypical frontotemporal lobar degeneration (FTLD) with fused in sarcoma inclusions (aFTLD-U) associated with *AP2A2* (Martins-De-Souza et al., 2012). This finding suggests that some AD candidate loci may also be involved in neurodegenerative disorders unrelated to AD pathogenesis. Indeed, there is some evidence in the literature suggesting the existence of cross-contamination of FTLD cases in AD datasets and *vice versa* (Hernández et al., 2014). The isolation of multiple genetic loci associated with other cognitive disorders will be necessary for deep evaluation of this possibility. This will be of particular importance for this genetic marker because there were a large proportion of missing values in our data (*n* = 152), probably due to technical problems during genotyping. Further studies are necessary to corroborate this preliminary finding.

This study had a number of limitations. Our results suggest that there are no major associations of AD risk genes and NEs measured in MCI subjects; however, this conclusion must be treated with considerable caution, given the important limitations of the present study. One limitation is the design of the study, which was a cross-sectional investigation of baseline data. As a consequence, longitudinal recording of cognitive decline may be a more sensitive way to detect the effects of AD risk loci. In addition, the sample size of MCI subjects in this study was modest compared with those in previous investigations focusing on AD (Lambert et al., 2013; Ruiz et al., 2014), which may explain the lack of significance of associations with SNPs, such as *CLU*, previously reported in our meta-analysis as potential markers for MCI to AD progression (Lacour et al., 2016). In this study, however, *CLU* showed only a tentative association signal in the whole MCI sample. Additionally, we were unable to measure sensitivity and specificity values because of the small sample sizes; nevertheless, compared with case controls status, QTLs can offer increased statistical power and thus have less rigorous sample size requirements (Shen et al., 2014). Another limitation of this study is related to the four stratified MCI phenotypes from the ACE dataset with a reduced sample size and also, the lack of validation of findings relating to these phenotypes in independent cohorts as a consequence of stratification. Validation was not possible because the clinical classification of MCI used for patients in the ACE dataset (Delis et al., 1991; Petersen et al., 1999; Lopez et al., 2003; Petersen, 2004; Espinosa et al., 2013, 2017) was not applied in other MCI cohorts; however, previously reported QTL analyses (Hu et al., 2011; Shen et al., 2014) with relatively modest sample sets and without validation series have also identified some significantly associated genetic markers. Another limitation is that the design of this study cannot prove that genetic risk factors may modulate specific cognitive functions. Future hypothesis-driven studies might assist to us and others to progress in that direction but the present study is observational and it cannot be proven the causality of the genes in the specific cognitive functions. Finally, control population stratification has not been calculated. However, the populations are homogeneous because they are Spanish population. Large GWAS efforts in the Spanish and German populations have demonstrated a limited stratification compared to other multi-ethnic and multi-country studies (Steffens et al., 2006; Gayán et al., 2008).

In summary, our data support the role of the *APOE* genotype in episodic verbal memory for aMCI phenotypes and the total MCI sample, consistent with previous studies, and fail to identify other associations with top low penetrance AD risk genes. Furthermore, we found that *HS3ST1* exhibited a near study-wide significant association with backward digits assessment of working memory function in individuals with the Pr-aMCI phenotype. Moreover, *AP2A2* was associated with language function (repetition) only for patients with the Pr-aMCI phenotype. Both of these pieces of evidence independently support the involvement of these loci in adult neurodegenerative disorders. Further studies in larger longitudinal MCI samples are now warranted to replicate these data, and disentangle the genetic factors that influence MCI NEs. Information on loci acting in different neurocognitive domains associated with MCI and longitudinal data available on conversion to AD or other dementias will be of relevance for fine mapping of risk loci and selection of entry points for drug development in AD.

## Author Contributions

AE contributed to study conception and design, organized study procedures, acquired data, analyzed and interpreted data, and wrote the first draft of the manuscript and assembled the article. BH-O contributed to the conception and design of heat maps and data analysis. SM-G acquired data and critically revised the manuscript. LK acquired the data, contributed to data analysis, and critically revised the first draft of the manuscript and the article. SH-H, IH, SW, HW, MR-R, AM, LV, AL, OR-G, CA, SG, MMa, MS-S, AS, GO, GM-R, AP, MI, SR, JK, OP, LF, MH, JW, TL, SR-H, LM, PC, MMa, SP, NA, IdR, AO, and MA acquired the data and critically revised the manuscript. SV supervised the statistical analysis of the data and critically revised the manuscript. MN, MW, and FJ acquired the data and critically revised the manuscript. LT and MB provided support in obtaining funding and data acquisition, and critically revised the manuscript. ALR and AGR conceptualized the study, obtained funding, supervised all phases of the study as principal investigators, provided support in data analyses and data interpretation, and critically revised the manuscript and the article. All authors have done final approval of the version to be published and they agree to be accountable for all aspects of the work in ensuring that questions related to the accuracy or integrity of any part of the work have been appropriately investigated and resolved.

## Conflict of Interest Statement

The authors declare that the research was conducted in the absence of any commercial or financial relationships that could be construed as a potential conflict of interest.

## Footnotes

1 http://www.r-project.org/

2 http://www.r-project.org/

## Supplementary Material

Figure S1 Amnestic/non-amnestic × probable/possible MCI flowchart. (Espinosa et al., 2013; Lopez et al., 2003; Petersen, 2004).

Figure S2 Pr-aMCI subtype Heat Map 1) Orientation; 2) Attention and working memory; 3) Processing speed and Executive functions; 4) Language; 5) Verbal Learning and Memory; 6) Praxis; 7) Visual gnosis; 8) Global Cognition; Global orientation, summary of Temporal + Spatial + Personal orientations; WAIS-III: Wechsler Adult Intelligence Scale, Third edition; SKT: Syndrome Kurz Test; 15-BNT: the abbreviated Boston Naming Test with 15 items; Verbal learning WMS-III = 1st+2nd+3rd+4thtrial scores; WMS-III: Wechsler Memory Scale, Third Edition.

Figure S3 Pr-aMCI-storage subtype Heat Map 1) Orientation; 2) Attention and working memory; 3) Processing speed and Executive functions; 4) Language; 5) Verbal Learning and Memory; 6) Praxis; 7) Visual gnosis; 8) Global Cognition; Global orientation, summary of Temporal + Spatial + Personal orientations; WAIS-III: Wechsler Adult Intelligence Scale, Third edition; SKT: Syndrome Kurz Test; 15-BNT: the abbreviated Boston Naming Test with 15 items; Verbal learning WMS-III = 1st+2nd+3rd+4thtrial scores; WMS-III: Wechsler Memory Scale, Third Edition.

Table S1 Comparison of demographic, clinical and APOE-e4 data among the stratified four MCI phenotypes from ACE dataset.

Table S2 **(A)** Comparison of demographic, clinical and APOE-e4 data between patients with aMCI and naMCI phenotypes from the AgeCoDe dataset. **(B)** Comparison of demographic, clinical and APOE-e4 data between patients with aMCI and naMCI phenotypes from the DCN dataset. **(C)** The ANCOVA NE scores between aMCI and naMCI groups from the AgeCoDe dataset. **(D)** The ANCOVA NE scores between aMCI and naMCI groups from the DCN dataset.

Table S3 The ANCOVA comparing NE scores among the stratified four MCI phenotypes from ACE dataset.

Table S4 Genetic effects on NE in the four stratified MCI phenotypes from ACE dataset.

Table S5 Major results of AD loci unlinked to APOE in MCI neurocognitive endophenotypes by the stratified four MCI phenotypes from ACE dataset.

Table S6 Major results of AD loci unlinked to APOE in MCI neurocognitive endophenotypes by the stratified five MCI phenotypes from ACE dataset.

Table S7 GROUP: MCI total sample.

Table S8 GROUP: amnestic MCI.

Table S9 GROUP: non-amnestic MCI.

Table S10 Supplementary information from the Pr-aMCI-storage subtype on ACE dataset.

DATA SHEET S1 LinearRegressionResults∖RegressionResults_AgeCoDe: all_MCI, mild cognitive impairment total sample; CERAD, the consortium to establish a registry for Alzheimer’s disease; DR, delayed recall from the CERAD; edu, adjusted by educational level; full, adjusted by age, gender, and educational level; unadj, unadjusted by age, gender, and educational level; IR, immediate recall from the CERAD; REC, recognition from the CERAD; VF, semantic verbal fluency (animals 1 minute) from the CERAD; Amn_MCI, amnestic mild cognitive impairment; NONAmn, non-amnestic mild cognitive impairment. LinearRegressionResults∖RegressionResults_DCN: DCN, The German Dementia Competence Network; all_MCI, mild cognitive impairment total sample; CERAD, the consortium to establish a registry for Alzheimer’s disease; bnt, Boston naming test 15- item version from the CERAD; edu, adjusted by educational level; full, adjusted by age, gender, and educational level ; unadj, unadjusted by age, gender, and educational level;cdt, clock drawing test; commands, commands item from the ADAS-Cog; conpraRECALL, constructional praxis recall from the CERAD; conpra, constructional praxis from the CERAD; idpra, ideational praxis from the ADAS-Cog; ori, orientation from the ADAS-Cog; rec, recognition from the CERAD; tmta, Trail Making Test (part A); tmtb, Trail Making Test (part B); vf, semantic verbal fluency (animals 1 minute) from the CERAD; wldr, delayed recall from the CERAD; wlir, immediate recall from the CERAD; wms_lmII, logical memory delayed recall of the story assessed according to the Wechsler memory scale; wms_lmI, logical memory immediate recall of the story from the Wechsler memory scale.

## Abbreviations

AD: Alzheimer’s disease
MCI: mild cognitive impairment
aMCI: amnestic MCI
naMCI: non-amnestic MCI
Pr-aMCI: probable amnestic MCI
Pr-naMCI: probable non-amnestic MCI
Pss-aMCI: possible amnestic MCI
Pss-naMCI: possible non-amnestic MCI
GWAS: genome-wide association studies
SNP: single-nucleotide polymorphism
NE: neurocognitive endophenotype
ACE: Fundació ACE
AgeCoDe: German study on Aging, Cognition, and Dementia in primary care patients
DCN: German Dementia Competence Network
*APOE*: apolipoprotein E
HS3ST1: heparan sulfate-glucosamine 3-sulfotransferase 1
AP2A2: adaptor-related protein complex 2 alpha 2 subunit
NBACE: neuropsychological battery of Fundació ACE
NBACE-L: learning on memory assessed according to WMS-III within NBACE
NBACE-DR: delayed recall on memory assessed according to WMS-III within NBACE
NBACE-RE: recognition on memory assessed according to WMS-III within NBACE
CERAD: the consortium to establish a registry for Alzheimer’s disease
CERAD-IR: CERAD-immediate recall
CERAD-DR: CERAD-delayed recall
CERAD-RE: CERAD-recognition
LM-IR: logical memory immediate recall assessed according to the Wechsler memory scale
LM-DR: logical memory delayed recall assessed according to the Wechsler memory scale
