# Supplementary material for: Exploring Genetic Associations of Alzheimer’s Disease Loci With Mild Cognitive Impairment Neurocognitive Endophenotypes
Source: Front Aging Neurosci. 2018 Oct 30;10:340. doi: 10.3389/fnagi.2018.00340 (PMC6218590; doi:10.3389/fnagi.2018.00340)
Supplement: Figure S1 — Amnestic/non-amnestic × probable/possible MCI flowchart. (Espinosa et al., 2013; Lopez et al., 2003; Petersen, 2004). [file Image_1.pdf]

Supplementary Figure S1. Amnestic/non-amnestic x probable/possible MCI flowchart. (Espinosa et al., 2013; Lopez et al., 2003; Petersen, 2004)

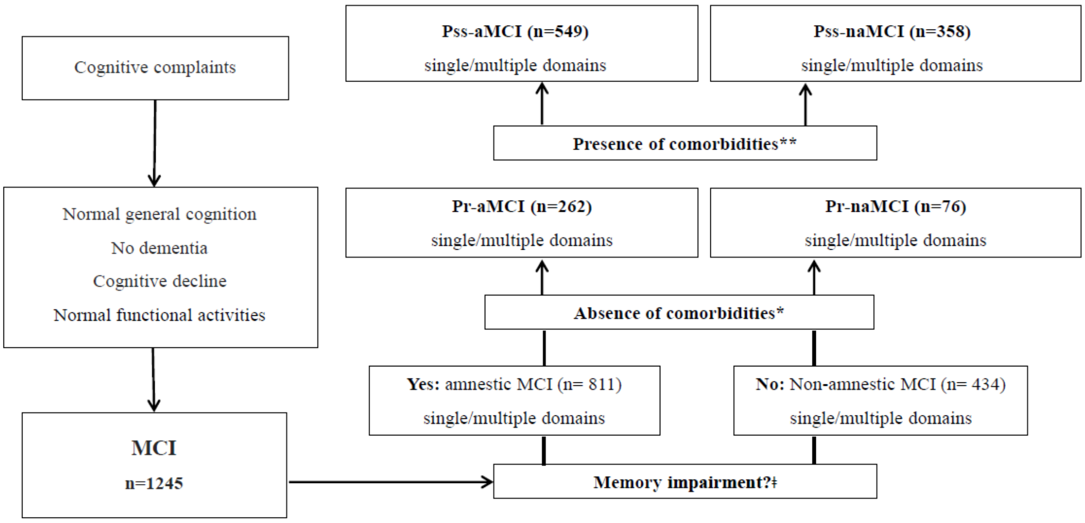

*: i.e., cerebrovascular disease, psychiatric disorders, history of head trauma encephalopathy, infectious diseases, or developmental disabilities that could themselves cause cognitive deficits;**: only when cerebrovascular disease, anxiety or depression was suspected; ǂ:measurable impairment in memory function, by TheWord List Learning test from the WMS-III with or without a deficit in other cognitive domains.
