# Supplementary figures and images for: Exploring Genetic Associations of Alzheimer’s Disease Loci With Mild Cognitive Impairment Neurocognitive Endophenotypes
Source: Front Aging Neurosci. 2018 Oct 30;10:340. doi: 10.3389/fnagi.2018.00340 (PMC6218590; doi:10.3389/fnagi.2018.00340)

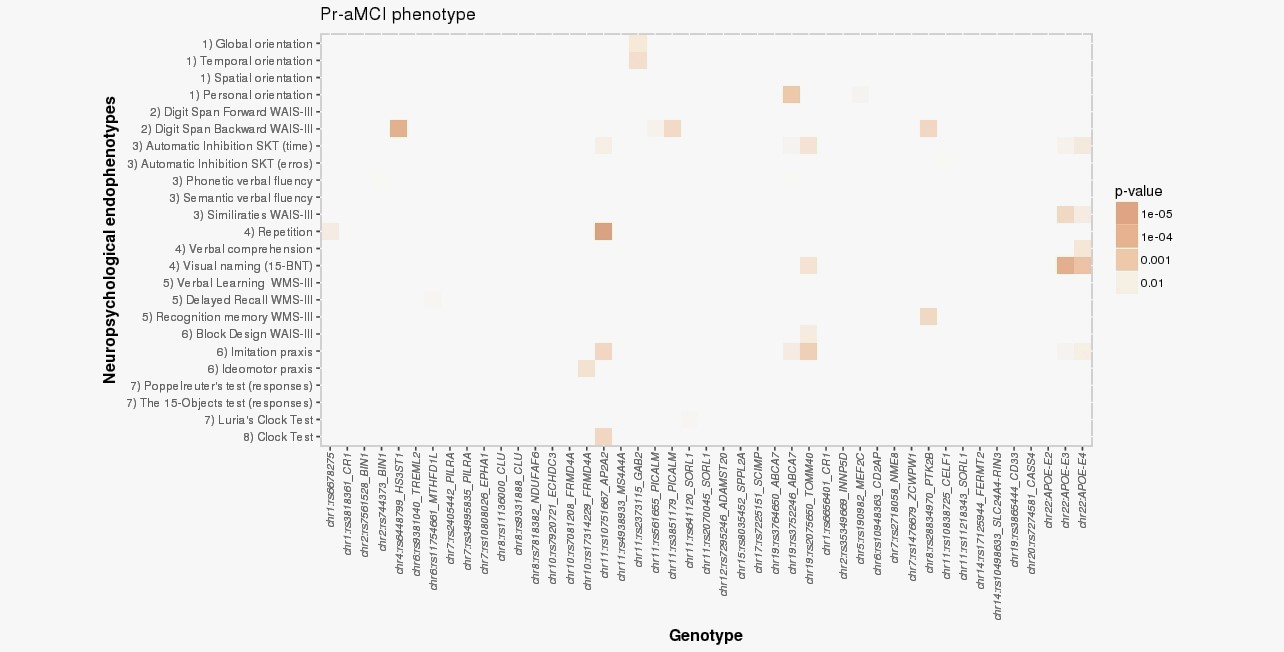

Supplement: Figure S2 — Pr-aMCI subtype Heat Map 1) Orientation; 2) Attention and working memory; 3) Processing speed and Executive functions; 4) Language; 5) Verbal Learning and Memory; 6) Praxis; 7) Visual gnosis; 8) Global Cognition; Global orientation, summary of Temporal + Spatial + Personal orientations; WAIS-III: Wechsler Adult Intelligence Scale, Third edition; SKT: Syndrome Kurz Test; 15-BNT: the abbreviated Boston Naming Test with 15 items; Verbal learning WMS-III = 1st+2nd+3rd+4thtrial scores; WMS-III: Wechsler Memory Scale, Third Edition. [file Image_2.jpeg]

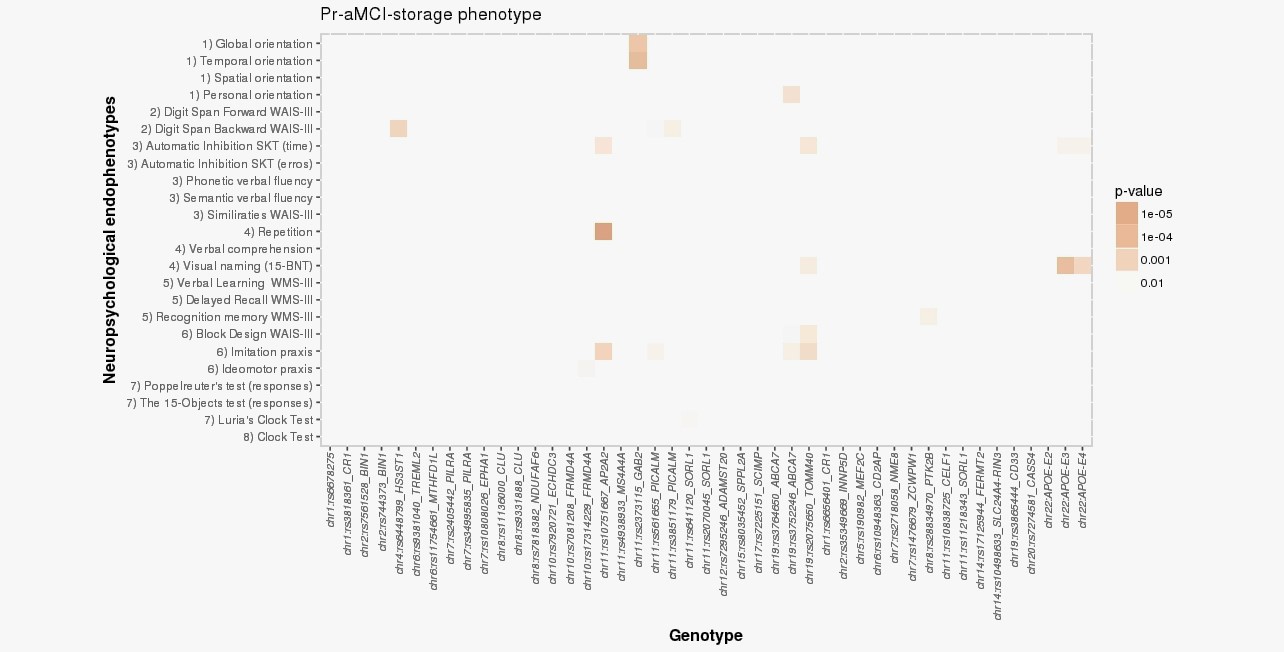

Supplement: Figure S3 — Pr-aMCI-storage subtype Heat Map 1) Orientation; 2) Attention and working memory; 3) Processing speed and Executive functions; 4) Language; 5) Verbal Learning and Memory; 6) Praxis; 7) Visual gnosis; 8) Global Cognition; Global orientation, summary of Temporal + Spatial + Personal orientations; WAIS-III: Wechsler Adult Intelligence Scale, Third edition; SKT: Syndrome Kurz Test; 15-BNT: the abbreviated Boston Naming Test with 15 items; Verbal learning WMS-III = 1st+2nd+3rd+4thtrial scores; WMS-III: Wechsler Memory Scale, Third Edition. [file Image_3.jpeg]
