# Supplementary material for: Exploring Genetic Associations of Alzheimer’s Disease Loci With Mild Cognitive Impairment Neurocognitive Endophenotypes
Source: Front Aging Neurosci. 2018 Oct 30;10:340. doi: 10.3389/fnagi.2018.00340 (PMC6218590; doi:10.3389/fnagi.2018.00340)
Supplement: Table S1 — Comparison of demographic, clinical and APOE-e4 data among the stratified four MCI phenotypes from ACE dataset. [file Table_1.DOCX]

|  | Pr-aMCI | Pss-aMCI | Pr-naMCI | Pss-naMCI | Statistics | p |
| --- | --- | --- | --- | --- | --- | --- |
| n (%) | 262 (21.0) | 549 (44.1) | 76 (6.1) | 358 (28.8) |  |  |
| Sex n (%) Female | 169 (64.5) | 354 (64.5) | 42 (55.3) | 245 (68.4) | 5.12_1_ | 0.163 |
| Education in years (n (%)) |  |  |  |  | 17.87_1_ | 0.001 |
| < 8 | 192 (73.3) | 446 (81.2) | 47 (61.8) | 280 (78.2) |  |  |
| > 8 | 70 (26.7) | 103 (18.8) | 29 (38.2) | 78 (21.8) |  |  |
| Age in years (mean/SD) | 76.5 / 6.4 | 76.2 / 7.3 | 76.3 / 5.7 | 75.2 / 7.4 | 2.21_2_ | 0.085 |
| MMSE (mean/SD) | 24.6 / 3.0 | 25.1 / 3.0 | 26.8 / 2.0 | 27.0 / 2.3 | 54.66_2_ | 0.001 |
| HIS (mean/SD) | 1.9 / 1.3 | 2.8 / 2.1 | 2.7 / 3.8 | 3.0 / 2.7 | 11.12_2_ | 0.001 |
| *APOE*-ε4 n (%) (presence of ε4 or ε4 ε4) | 119 (45.4) | 175 (32.0) | 15 (19.7) | 94 (26.3) | 32.04_1_ | 0.001 |

**Supplementary Table S1. Comparison of demographic, clinical and *APOE-*ε4 data among the stratified four MCI phenotypes from ACE dataset**

Pr-aMCI: Probable amnestic Mild Cognitive Impairment; Pss-aMCI: Possible amnestic Mild Cognitive Impairment; Pr-naMCI: Probable non-amnestic Mild Cognitive Impairment; Pss-naMCI: Possible non-amnestic Mild Cognitive Impairment; MMSE: Mini-Mental State Examination; HIS: Hachinski Ischemia Scale; *APOE*: Apolipoprotein E; SD: Standard deviation; _1_: χ^2^; _2_: F.
