# Supplementary material for: Exploring Genetic Associations of Alzheimer’s Disease Loci With Mild Cognitive Impairment Neurocognitive Endophenotypes
Source: Front Aging Neurosci. 2018 Oct 30;10:340. doi: 10.3389/fnagi.2018.00340 (PMC6218590; doi:10.3389/fnagi.2018.00340)
Supplement: Table S3 — The ANCOVA comparing NE scores among the stratified four MCI phenotypes from ACE dataset. [file Table_3.DOCX]

| **Supplementary Table S3. The ANCOVA comparing NE scores among the stratified four MCI phenotypes from ACE dataset** | | | | | | | | | |
| --- | --- | --- | --- | --- | --- | --- | --- | --- | --- |
| **NE on NBACE** | Pr-aMCI  M (SD) | Pss-aMCI  M (SD) | Pr-naMCI  M (SD) | Pss-naMCI  M (SD) | F (3, 537) | Eta^2^ | Multiple Comparisons with Bonferroni’s correction  Pr-aMCI vs.  Pss-aMCI Pr-naMCI Pss-naMCI | | |
| **Global orientation** | 13.60 (1.66) | 13.88 (1.44) | 14.16 (1.23) | 14.64 (.78) | 37.41*** | 0.08 | 0.032 | 0.007 | 0.001 |
| **Attention and working memory** | | | | | | | | | |
| Forward Digits | 6.63 (1.74) | 6.57 (1.68) | 6.83 (1.55) | 6.84 (1.72) | 2.28 | 0.01 | NS | NS | NS |
| Backward Digits | 3.36 (1.52) | 3.14 (1.50) | 3.46 (1.66) | 3.71 (1.55) | 11.88* | 0.03 | NS | NS | 0.015 |
| **Executive functions** | | | | | | | | | |
| SKT (time in seconds) | 42.90 (16.38) | 46.42 (22.40) | 42.28 (20.07) | 39.04 (13.34) | 11.74 | 0.03 | NS | NS | NS |
| SKT (errors) | 4.69 (5.35) | 4.59 (5.17) | 3.37 (3.86) | 3.00 (3.72) | 10.49*** | 0.03 | NS | NS | 0.001 |
| PVF | 9.01 (4.40) | 8.14 (4.04) | 9.84 (4.45) | 10.03 (4.37) | 16.75* | 0.04 | 0.030 | NS | 0.013 |
| SVF | 10.89 (3.97) | 11.18 (3.77) | 12.55 (4.21) | 13.50 (4.16) | 32.78*** | 0.07 | NS | 0.007 | 0.001 |
| Similarities WAIS-III | 7.64 (3.18) | 7.95 (2.88) | 9.04 (2.86) | 9.00 (2.60) | 18.21*** | 0.04 | NS | 0.001 | 0.001 |
| **Language** | | | | | | | | | |
| Visual naming (15-BNT) | 12.33 (2.67) | 12.69 (2.30) | 13.33 (1.62) | 13.55 (1.77) | 19.50*** | .045 | NS | 0.003 | 0.001 |
| **Verbal Learning and Memory WMS-III** | | | | | | | | | |
| Learning ( Trials 1+2+3+4) | 15.51 (5.14) | 16.84 (4.72) | 22.08 (5.11) | 23.53 (5.04) | 199.90*** | 0.33 | 0.001 | 0.001 | 0.001 |
| Delayed Recall | 0.85 (1.14) | 1.59 (1.50) | 4.80 (1.73) | 5.33 (1.77) | 627.76*** | 0.60 | 0.001 | 0.001 | 0.001 |
| Recognition memory | 17.72 (2.88) | 19.10 (2.56) | 21.88 (1.58) | 21.99 (1.65) | 209.38 *** | 0.34 | 0.001 | 0.001 | 0.001 |
| **Praxis** | | | | | | | | | |
| Block Design | 2.63 (1.38) | 2.64 (1.39) | 3.07 (1.14) | 3.03 (1.21) | 8.71*** | 0.02 | NS | NS | 0.001 |
| Imitation | 2.81 (1.25) | 2.89 (1.21) | 3.20 (1.05) | 3.27 (1.04) | 11.03*** | 0.03 | NS | NS | 0.001 |
| **Visual gnosis** | | | | | | | | | |
| Poppelreuter test (responses) | 8.72 (1.55) | 8.87 (8.85) | 9.27 (1.00) | 9.31 (1.09) | 13.02*** | 0.03 | NS | 0.008 | 0.001 |
| Luria’s Clocks test | 2.43 (1.26) | 2.54 (1.21) | 2.97 (1.04) | 2.83 (1.04) | 9.35*** | 0.02 | NS | 0.002 | 0.001 |
| The 15-Objects test | 10.70 (2.45) | 10.79 (2.77) | 11.40 (2.06) | 11.48 (2.07) | 1.70 | 0.02 | NS | NS | NS |
| **Global Cognition** | 4.56 (2.28) | 4.90 (2.11) | 5.48 (1.78) | 5.71 (1.75) | 20.11*** | 0.05 | NS | 0.004 | 0.001 |

NE: Neurocognitive Endophenotypes; NBACE: neuropsychological battery of Fundació ACE; Pr-aMCI: Probable amnestic Mild Cognitive Impairment; Pr-naMCI: Probable non-amnestic Mild Cognitive Impairment; Pss-aMCI: Possible amnestic Mild Cognitive Impairment; Pss-naMCI: Possible non-amnestic Mild Cognitive Impairment; NBACE: neuropsychological battery of Fundació ACE; Global orientation: summatory of Temporal+Spatial+Personal orientations; WMS-III: Wechsler Memory Scale, Third Edition; The abbreviated BNT: Boston Naming Test with 15 visual items; Recognition memory: correct answers; Block Design: WAIS-III; SKT: Automatic Inhibition Symdrom Kurztest; PVF: Phonemic verbal fluency; SVF: Semantic verbal fluency ; WAIS-III: Weschler Adult Intelligence Scale, Third edition; Global Cognition: Clock Test; M: Mean; S.D: Standard deviation; NS: p≥ .05; *p< .05, **p≤ .005, ***p≤ .001.
