# Supplementary material for: Exploring Genetic Associations of Alzheimer’s Disease Loci With Mild Cognitive Impairment Neurocognitive Endophenotypes
Source: Front Aging Neurosci. 2018 Oct 30;10:340. doi: 10.3389/fnagi.2018.00340 (PMC6218590; doi:10.3389/fnagi.2018.00340)
Supplement: Table S4 — Genetic effects on NE in the four stratified MCI phenotypes from ACE dataset. [file Table_4.DOCX]

| **MCI**  **group** | **4A Pr-aMCI** | | | **4B Pss-aMCI** | | | **4C Pr-naMCI** | | | **4D Pss-naMCI** | | |
| --- | --- | --- | --- | --- | --- | --- | --- | --- | --- | --- | --- | --- |
| **NE** | **NBACE-L** | **NBACE-DR** | **NBACE-RE** | **NBACE-L** | **NBACE-DR** | **NBACE-RE** | **NBACE-L** | **NBACE-DR** | **NBACE-RE** | **NBACE-L** | **NBACE-DR** | **NBACE-RE** |
| **n** | 262 | 262 | 262 | 549 | 549 | 549 | 76 | 76 | 76 | 358 | 358 | 358 |
| **β** | 0.42 | -0.11 | 0.17 | -1.37 | -0.36 | -0.43 | .29 | -0.33 | .16 | -0.30 | -0.24 | 0.17 |
| **L95/U95** | -0.59/1.43 | -0.34/0.12 | -0.42/0.75 | -2.07/-0.68 | -0.59/-0.12 | -0.85/-0.02 | -1.86/2.42 | -1.02/0.36 | -0.54/0.85 | -1.38/0.78 | -0.61/0.13 | -0.21/0.55 |
| **p-value** | 0.397 | 0.328 | 0.561 | 5.82x10^-5^*** | 0.002 | 0.031 | 0.785 | 0.323 | 0.646 | 0.578 | 0.191 | 0.357 |

**Supplementary Table S4. Genetic effects on NE in the four stratified MCI phenotypes from ACE dataset**

NE: Neurocognitive endophenotypes; NBACE: neuropsychological battery of Fundació ACE; Pr-aMCI: Probable amnestic Mild Cognitive Impairment; Pr-naMCI: Probable non-amnestic Mild Cognitive Impairment; Pss-aMCI: Possible amnestic Mild Cognitive Impairment; Pss-naMCI: Possible non-amnestic Mild Cognitive Impairment; NBACE- L: Learning on Memory of the WMS-III from NBACE; NBACE- DR: Delayed Recall on Memory of the WMS-III from NBACE; NBACE-RE: Recognition on Memory of the WMS-III from NBACE; β: unstandardized regression coefficient; L-U95: confidence intervals 95% ; *Statistically significant after Bonferroni’s correction (*p*≤ 1.02 *10-E^-5^*).
