# Supplementary material for: Exploring Genetic Associations of Alzheimer’s Disease Loci With Mild Cognitive Impairment Neurocognitive Endophenotypes
Source: Front Aging Neurosci. 2018 Oct 30;10:340. doi: 10.3389/fnagi.2018.00340 (PMC6218590; doi:10.3389/fnagi.2018.00340)
Supplement: Table S5 — Major results of AD loci unlinked to APOE in MCI neurocognitive endophenotypes by the stratified four MCI phenotypes from ACE dataset. [file Table_5.DOCX]

**Supplementary Table S5. Major results of AD loci unlinked to *APOE* in MCI neurocognitive endophenotypes by the stratified four MCI phenotypes from ACE dataset**

| **NE** | **n** | **Locus/ snp** | **β** | **L95** | **U95** | **p-value** |
| --- | --- | --- | --- | --- | --- | --- |
| **Pr-aMCI** | | | | | | |
| **NBACE-Backward Digits** | 262 | ***HS3ST1***-**rs6448799** | 0.52 | 0.25 | 0.78 | 7.57x10^-5^*** |
| **NBACE-Repetition** | 110 | ***AP2A2-*rs10751667** | -0.19 | -0.27 | -0.11 | 5.34x10^-6^*** |

NE: Neurocognitive endophenotypes; NBACE: neuropsychological battery of Fundació ACE; β: Beta; L-U95: confidence intervals 95%; *Statistically significant after Bonferroni’s correction (*p* ≤ 1.02 *10-E^-5^*).
