# Supplementary material for: Exploring Genetic Associations of Alzheimer’s Disease Loci With Mild Cognitive Impairment Neurocognitive Endophenotypes
Source: Front Aging Neurosci. 2018 Oct 30;10:340. doi: 10.3389/fnagi.2018.00340 (PMC6218590; doi:10.3389/fnagi.2018.00340)
Supplement: Table S6 — Major results of AD loci unlinked to APOE in MCI neurocognitive endophenotypes by the stratified five MCI phenotypes from ACE dataset. [file Table_6.DOCX]

**Supplementary Table S6. Major results of AD loci unlinked to *APOE* in MCI neurocognitive endophenotypes by the stratified five MCI phenotypes from ACE dataset**

| **Pr-aMCI-storage subtype** | | | | | | |
| --- | --- | --- | --- | --- | --- | --- |
| Backward Digits | 216 | *HS3ST1*-rs6448799 | 0.27 | 0.10 | 0.45 | 0.001657 |
| Repetition | 94 | *AP2A2-*rs10751667 | -0.24 | -0.33 | -0.15 | 1.165E-06*** |

β: Beta; L-U95: confidence intervals 95% ; *Statistically significant after Bonferroni’s correction (*p* ≤*10-E^-5^*).
