# Supplementary material for: Exploring Genetic Associations of Alzheimer’s Disease Loci With Mild Cognitive Impairment Neurocognitive Endophenotypes
Source: Front Aging Neurosci. 2018 Oct 30;10:340. doi: 10.3389/fnagi.2018.00340 (PMC6218590; doi:10.3389/fnagi.2018.00340)
Supplement: Table S10 — Supplementary information from the Pr-aMCI-storage subtype on ACE dataset. [file Table_10.docx]

## Supplementary information from the Pr-aMCI-storage subtype on ACE dataset

A multivariate linear regression analysis adjusted by age, gender, and educational level was carried out among every SNP and the NEs using PLINK software (v. 1.07) (Purcell et al., 2007). This procedure was performed for the MCI stratified into five subtypes (Pr-aMCI, Pss-aMCI, Pss-naMCI, Pr-naMCI and Pr-aMCI-storage subtype). PLINK software (v. 1.07) (Purcell et al., 2007) was used to analyze genetic variation by calculating the allele frequencies in our populations, the polymorphism information content, the Hardy-Weinberg equilibrium, and deviations in allele frequencies between the study groups, using the Chi-square or Fisher’s exact test, and linkage disequilibrium (LD) with flanking markers (when needed). Data are graphically represented using heat maps, where individual p-values contained in a matrix are represented by color-coding indicating the statistical significance values calculated for SNPs and hierarchical NEs using R (The Project R for Statistical Informatics software) (v. 3.3.1) (<http://www.r-project.org/>). Further, Bonferroni’s correction was executed to adjust for multiple testing. This procedure was performed for both of the following:

The MCI stratified into five subtypes as follows:

0.05/(6 MCI phenotype-related conditions (including MCI total sample) × 24 NEs × 41 SNPs (including *APOE*)) = 8.49 10-E^-6^.

Thus, only observed associations below p ≤ 10^-6^ were considered significantly associated. A trend towards association was assumed at p ≤ 10^-5^. Finally, significant associations in the MCI stratified into four subtypes were not replicated in AgeCoDe and DCN datasets because probable/possible MCI stratifications were not available in these cohorts.
